# Disease-Free Survival after Hepatic Resection in Hepatocellular Carcinoma Patients: A Prediction Approach Using Artificial Neural Network

**DOI:** 10.1371/journal.pone.0029179

**Published:** 2012-01-03

**Authors:** Wen-Hsien Ho, King-Teh Lee, Hong-Yaw Chen, Te-Wei Ho, Herng-Chia Chiu

**Affiliations:** 1 Department of Healthcare Administration and Medical Informatics, Kaohsiung Medical University, Kaohsiung, Taiwan; 2 Department of Surgery, Kaohsiung Medical University Hospital, Kaohsiung, Taiwan; 3 Yuan's Hospital, Kaohsiung, Taiwan; 4 Department of Health, Bureau of Health Promotion, Taipei, Taiwan; Technische Universität München, Germany

## Abstract

**Background:**

A database for hepatocellular carcinoma (HCC) patients who had received hepatic resection was used to develop prediction models for 1-, 3- and 5-year disease-free survival based on a set of clinical parameters for this patient group.

**Methods:**

The three prediction models included an artificial neural network (ANN) model, a logistic regression (LR) model, and a decision tree (DT) model. Data for 427, 354 and 297 HCC patients with histories of 1-, 3- and 5-year disease-free survival after hepatic resection, respectively, were extracted from the HCC patient database. From each of the three groups, 80% of the cases (342, 283 and 238 cases of 1-, 3- and 5-year disease-free survival, respectively) were selected to provide training data for the prediction models. The remaining 20% of cases in each group (85, 71 and 59 cases in the three respective groups) were assigned to validation groups for performance comparisons of the three models. Area under receiver operating characteristics curve (AUROC) was used as the performance index for evaluating the three models.

**Conclusions:**

The ANN model outperformed the LR and DT models in terms of prediction accuracy. This study demonstrated the feasibility of using ANNs in medical decision support systems for predicting disease-free survival based on clinical databases in HCC patients who have received hepatic resection.

## Introduction

Globally, hepatocellular carcinoma (HCC) is among the most prevalent malignant tumors [1]. Of all cancers, HCC has had the highest and second highest mortality rates in males and in females, respectively, since the early 1980s [2]. In Taiwan, the incidence rates of HCC have steadily increased in the past two decades: the respective age-standardized incidence rates for men and women increased from 55.8 and 22.3 per 100,000 in 2002 to 62.1 and 25.6 per 100,000 in 2007 [3]. In 2009, HCC also comprised 38.0% and 14.9% of all cancer-related deaths in men and women in Taiwan, respectively [4]. Hepatic resection is the most common treatment modality for HCC and is among the most effective interventions [5]–[7] for achieving long-term survival. However, even after undergoing hepatic resection, patients with HCC may still have very poor prognoses because of the low survival and high recurrence rates associated with this procedure [8]. Therefore, the aim of this study was to construct an accurate and effective model for predicting disease-free survival in HCC patients who have received hepatic resection. An improved model would enable further development of computerized medical decision support systems for aiding surgeons and healthcare institutions in constructing guidelines for interpreting clinical outcomes. Although previous studies [9], [10] have examined disease-free survival rates at various endpoints, none have evaluated the accuracy of models for predicting disease-free survival after hepatic resection in HCC patients at different endpoints (i.e., 1, 3, and 5 years after resection).

Recently, machine-learning and statistical methods have been applied to develop prediction models for clinical diagnosis and treatment, e.g., artificial neural networks (ANNs), logistic regression (LR) and decision tree (DT) (see, e.g., [11]–[27] and the references therein). Clinical application of these prediction models can potentially improve diagnostic accuracy, treatment decisions, and efficiency in using limited health care resources [11].

Artificial neural networks have proven particularly effective for nonlinear mapping based on human knowledge and are attracting interest for use in solving complex classification problems [28], [29]. A multilayer ANN containing layers of simple computing nodes is analogous to brain neural networks that can accurately approximate nonlinear continuous functions and reveal previously unknown relationships between given input and output variables [30], [31]. Because of their unique structure, ANNs can learn by using algorithms such as backpropagation algorithm and evolutionary algorithm [32], [33]. Potential medical applications of ANNs include problems in which the relationship between independent variables and clinical outcome are poorly understood [34]. Because ANNs are capable of self training with minimal human intervention, many studies of large epidemiology databases have, in addition to traditional statistical methods, used ANNs for further insight into the interrelationships among variables. However, since few studies have compared performance between ANNs and other modeling techniques such as LR and DT, these interrelationships are still unclear [35]. Our objective was to fill a gap in the current literature by comparing the predictive performance of three modeling techniques so that improved models for predicting 1-, 3- and 5-year disease-free survival can be implemented in knowledge-based computer programs and in medical decision support systems.

This study therefore constructed a database of HCC patients who had received hepatic resection between 2000 and 2007 at either of two hospitals in Kaohsiung, Taiwan: Kaohsiung Medical University Hospital and Yuan's Hospital. The database included demographic, clinical, surgical and outcome data. An ANN model, an LR model, and a DT model were constructed to predict 1-, 3- and 5-year disease-free survival. The three models were based on data for 80% of the cases, which were randomly selected. The remaining 20% of the cases were then used for performance tests of the three models. Predictive accuracy was compared by areas under receiver operating characteristics curve (AUROC) analyses.

## Methods

### Data collection and variable selection

The study population included 482 patients who had received liver resection for HCC and were currently disease-free. The exclusion criteria were any history of the following: (i) liver resection; (ii) treatment with radiofrequency ablation or microwave ablation; (iii) histopathological evidence of benign tumor and/or non-primary liver cancer; (iv) unavailable and/or incomplete medical history; (v) death within thirty days after surgery; (vi) tumor remaining after resection; (vii) incomplete data for key explained variables; and (viii) follow-up data for less than 1 year. Therefore, 427, 354 and 297 patients were classified into the 1-, 3- and 5-year disease-free survival groups, respectively. In each patient, medical records were reviewed by the attending physician. Data collection included demographic data, clinical features, and surgical process and outcome. Ethical approval was provided by Institutional Review Board of the Kaohsiung Medical University Chung-Ho Memorial Hospital (KMUH-IRB-990166). Patients provided written informed consent.

Patients were classified as disease-free hepatic resection survivors if no death or recurrence occurred during the 1-, 3-, or 5-year periods considered in the three survival models. In other words, survival (no event) was defined as disease-free survival after 1, 3, or 5 years. Therefore, presence of an event (death or recurrence) was coded as 1, and absence of an event (disease-free survival) was coded as 0.

First, continuous explanatory variables were transformed into categorical variables to minimize the effects of extreme values and to enhance the computing efficiency of the ANN model. The cut-off points for these variables were based on those used in previous clinical studies [5], [7], [36]–[40]. Low and high risk were coded as 0 and 1, respectively. The variables included BUN AST, α-fetoprotein, ALT, total bilirubin, and others. Other recoded items included TNM stage, a common prognostic index of cancer risk or severity, and ASA, a risk score for surgical procedures. The TNM stage ranges from 1 to 6, and ASA score ranges from 1 to 4. Two variables were recoded as 0 for low risk, 1 for medium risk, and 2 for high risk (Table 1). High risk was assumed to increase the probability of recurrence (event). Second, to enhance the calculation efficiency and prediction performance of the ANN models, univariate Cox proportional hazard model was used to test relationships among potential variables. Variables with statistically significant associations (log-rank test, P<0.05) with disease-free survival were retained to construct the ANN models (Table 1). Finally, of the 31 input variables, the 15 statistically significant variables used to construct the ANN models were liver cirrhosis, chronic hepatitis, AST, ALT, total bilirubin, albumin, creatinine, ASA classification, Child-Pugh classification, TNM stage, tumor number, portal vein invasion, biliary invasion, surgical procedure, and post-operative complication. Age and gender were also included as control variables.

**Table 1:** Potential input variables for prediction models (N = 482).

Variables	Value	P value
Demographic characteristics		
Age (years)^a^	0:⩽65, 1:>65 (mean = 57.7)	0.43
Gender^a^	0: male, 1: female	0.43
Clinical features		
Comorbidity	0: no, 1: yes	0.16
Liver cirrhosis^b^	0: no, 1: yes	<0.001
Chronic hepatitis^b^	0: no, 1: HBV, 2: HCV, 3: HBCV	0.29, 0.02, 0.01
α-Fetoprotein (ng/ml)	0:⩽100, 1:>100	0.10
AST (U/L)^b^	0:⩽80, 1:>80	<0.001
ALT (U/L)^b^	0:⩽80, 1:>80	<0.001
Total bilirubin (mg/dl)^b^	0:⩽1.0, 1:>1.0	0.01
Albumin (g/dl)^b^	0:>3.5, 1:⩽3.5	<0.001
BUN (mg/dl)	0:⩽21, 1:>21	0.44
Creatinine (mg/dl)^b^	0:⩽1.4, 1:>1.4	0.09
Platelet (10^3^/µl)	0:>150, 1:⩽150	<0.001
Prothrombin time (%)	0:⩽80, 1:>80	0.61
ICGR_15_ (%)	0:⩽15, 1:>15	0.15
ASA classification^b^	0: ASA = 1, 1: ASA = 2, 2: ASA = 3, 4	0.01, 0.13
Child-Pugh classification^b^	0: A, 1: B,C	0.01
TNM stage^b^	0: I, 1: II, 2: IIIa, IIIb, IIIc, IV	<0.001, <0.001
Tumor number^b^	0: single, 1: multiple	<0.001
Tumor size (cm)	0:⩽5, 1:>5	0.08
Portal vein invasion^b^	0: no, 1: yes	<0.001
Biliary invasion^b^	0: no, 1: yes	0.02
Surgical process and outcome		
Surgical procedure^b^	0: laparoscopic, 1: open surgery	<0.001
Extent of resection	0: minor, 1: major	0.45
Resection margin (mm)	0:>10, 1:⩽10	0.15
Surgical time	0:⩽180, 1:>180	0.34
Blood loss (ml)	0:⩽1000, 1:>1000	0.71
Blood transfusion	0: no, 1: yes	0.65
Blood transfusion (ml)	0:⩽1000, 1:>1000	0.06
Post-operative complication^b^	0: no, 1: yes	0.01
Preoperative treatment	0: no, 1: yes	0.08

a : control input variable.

b : significant input variable.

### Training and validation data sets

From each of the three survival groups, 80% of the cases were assigned to training groups for developing the ANN, LR and DT models, and the remaining 20% were assigned to validation groups for performance tests of the models for predicting 1-, 3-, and 5-year disease-free survival. That is, of the 427 1-year cases, 342 were used for training, and 85 were used for validation; of the 354 3-year cases, 283 were used for training, and 71 were used for validation; of the 297 5-year cases, 238 were used for training, and 59 were used for validation (Table 2). Table 2 shows that (i) the specific data contained in each clinical case were summarized with their descriptive characteristics for 1-, 3-, and 5-year disease-free survival. For example, 245 (71.6%) patients were aged older than 65 years and 97 (28.4%) patients were aged 65 years or younger. In the 1-year training group, 252 (73.7%) patients were male, and 90 (26.3%) patients were female; (ii) at 1-, 3-, and 5 years after the resection procedure, post-resection events (i.e., recurrence or death) had occurred in 155 (36.3%), 226 (63.8%) and 247 (83.2%) patients; and (iii) in all three survival models, the effects of input variables did not significantly differ between training and validation (P>0.05), which confirmed the reliability of the data selection.

**Table 2:** Comparison of clinical features between training and validation groups.

Variables	Definitions	1-year(N = 427)					3-year(N = 354)					5-year(N = 297)				
		Training(N = 342)		Validation(N = 85)		P	Training(N = 283)		Validation(N = 71)		P	Training(N = 238)		Validation(N = 59)		P
		N	%	N	%		N	%	N	%		N	%	N	%	
Age	⩽65	245	71.6	67	78.8	0.181	206	72.8	54	76.1	0.578	177	74.4	40	67.8	0.308
	>65	97	28.4	18	21.2		77	27.2	17	23.9		61	25.6	19	32.2	
Gender	Male	252	73.7	70	82.4	0.097	214	75.6	53	74.6	0.865	175	73.5	45	76.3	0.667
	Female	90	26.3	15	17.6		69	24.4	18	25.4		63	26.5	14	23.7	
Liver cirrhosis	No	112	32.7	37	43.5	0.062	101	35.7	18	25.4	0.099	72	30.3	21	35.6	0.428
	Yes	230	67.3	48	56.5		182	64.3	53	74.6		166	69.7	38	64.4	
Chronic hepatitis	No	37	10.8	12	14.1	0.644	28	9.9	10	14.1	0.390	22	9.2	9	15.3	0.603
	HBV	185	54.1	40	47.1		145	51.2	35	49.3		119	50.0	27	45.8	
	HCV	95	27.8	27	31.8		90	31.8	18	25.4		75	31.5	18	30.5	
	HBCV	25	7.3	6	7.1		20	7.1	8	11.3		22	9.2	5	8.5	
AST	⩽80	284	83.0	65	76.5	0.161	227	80.2	56	78.9	0.801	185	77.7	47	79.7	0.748
	>80	58	17.0	20	23.5		56	19.8	15	21.1		53	22.3	12	20.3	
ALT	⩽80	272	79.5	65	76.5	0.536	217	76.7	57	80.3	0.516	178	74.8	48	81.4	0.290
	>80	70	20.5	20	23.5		66	23.3	14	19.7		60	25.2	11	18.6	
Total bilirubin	⩽1.0	246	71.9	63	74.1	0.686	203	71.7	53	74.6	0.623	166	69.7	46	78.0	0.211
	>1.0	96	28.1	22	25.9		80	28.3	18	25.4		72	30.3	13	22.0	
Albumin	>3.5	272	79.5	66	77.6	0.702	220	77.7	55	77.5	0.960	180	75.6	45	76.3	0.918
	⩽3.5	70	20.5	19	22.4		63	22.3	16	22.5		58	24.4	14	23.7	
Platelet	>150	169	49.4	44	51.8	0.698	130	45.9	39	54.9	0.175	107	45.0	31	52.5	0.296
	⩽150	173	50.6	41	48.2		153	54.1	32	45.1		131	55.0	28	47.5	
ASA Classification	1	90	26.3	12	14.1	0.062	79	27.9	17	23.9	0.700	67	28.2	21	35.6	0.387
	2	175	51.2	51	60.0		144	50.9	40	56.3		124	52.1	25	42.4	
	3, 4	77	22.5	22	25.9		60	21.2	14	19.7		47	19.7	13	22.0	
Child-Pugh Classification	A	334	97.7	83	97.6	0.994	277	97.9	68	95.8	0.314	230	96.6	58	98.3	0.504
	B, C	8	2.3	2	2.4		6	2.1	3	4.2		8	3.4	1	1.7	
TNM Stage	I	200	58.5	47	55.3	0.807	159	56.2	36	50.7	0.468	124	52.1	28	47.5	0.765
	II	108	31.6	30	35.3		94	33.2	29	40.8		88	37.0	23	39.0	
	IIIa, IIIb, IIIc, IV	34	9.9	8	9.4		30	10.6	6	8.5		26	10.9	8	13.6	
Tumor no.	Single	244	71.3	61	71.8	0.939	201	71.0	44	62.0	0.140	156	65.5	40	67.8	0.744
	Multiple	98	28.7	24	28.2		82	29.0	27	38.0		82	34.5	19	32.2	
Tumor size (cm)	⩽5	268	77.2	67	77.0	0.965	204	74.7	50	73.5	0.840	154	73.0	37	69.8	0.644
	>5	79	22.8	20	23.0		69	25.3	18	26.5		57	27.0	16	30.2	
Portal vein invasion	No	277	81.0	65	76.5	0.350	227	80.2	56	78.9	0.801	184	77.3	47	79.7	0.698
	Yes	65	19.0	20	23.5		56	19.8	15	21.1		54	22.7	12	20.3	
Biliary invasion	No	334	97.7	83	97.6	0.994	276	97.5	69	97.2	0.869	230	96.6	58	98.3	0.504
	Yes	8	2.3	2	2.4		7	2.5	2	2.8		8	3.4	1	1.7	
Surgical procedure	Laparoscopic	66	19.3	18	21.2	0.697	60	21.2	13	18.3	0.590	57	23.9	11	18.6	0.385
	Open surgery	276	80.7	67	78.8		223	78.8	58	81.7		181	76.1	48	81.4	
Post-operative complication	No	311	90.9	78	91.8	0.810	255	90.1	63	88.7	0.732	214	89.9	50	84.7	0.258
	Yes	31	9.1	7	8.2		28	9.9	8	11.3		24	10.1	9	15.3	
Disease-free survival status	No	211	61.7	61	71.8	0.084	108	38.2	20	28.2	0.117	36	15.1	14	23.7	0.114
	Yes	131	38.3	24	28.2		175	61.8	51	71.8		202	84.9	45	76.3	

### Modeling tools

**Figure 1:**
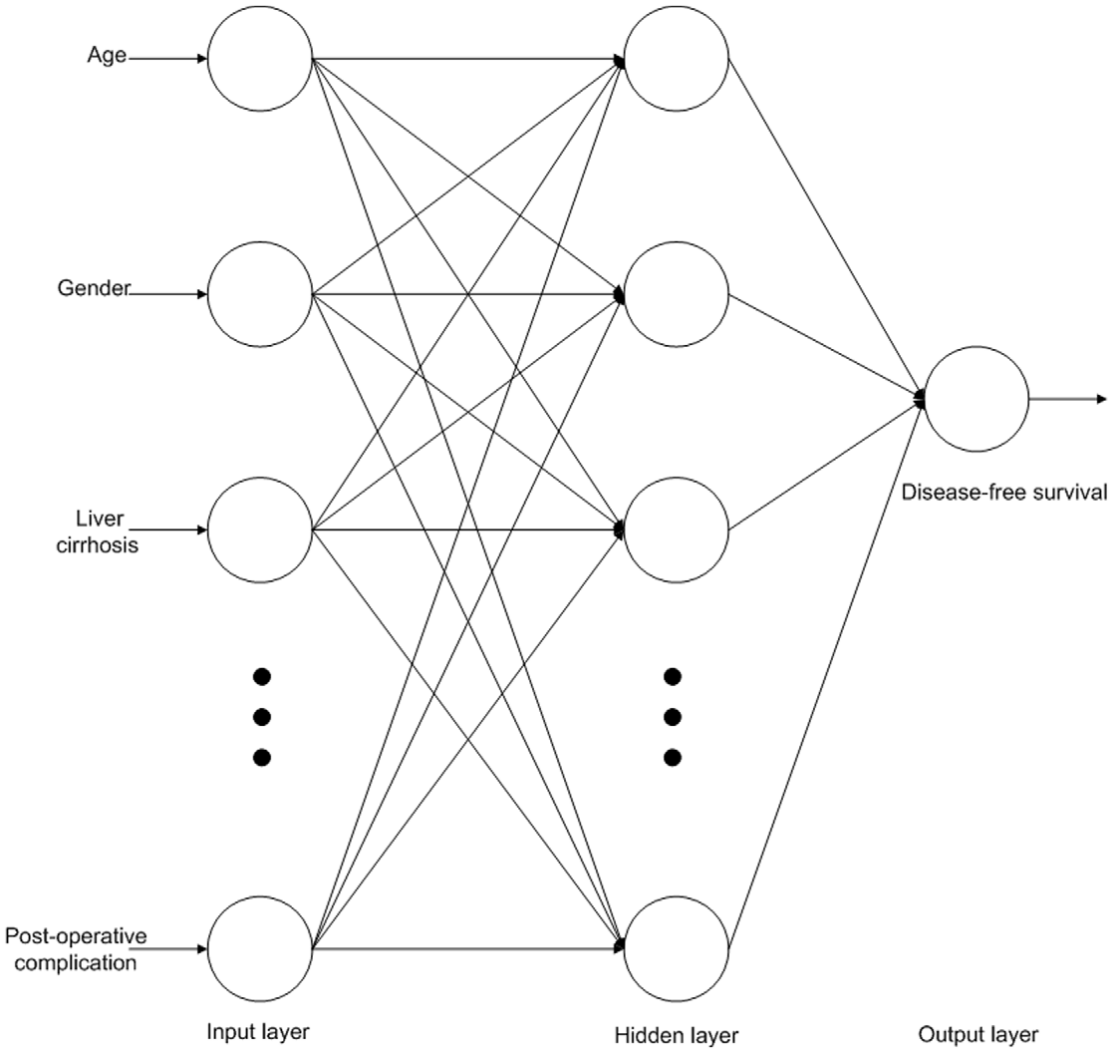
Framework of artificial neural network for the 1-, 3- and 5-year disease-free survival models. The input layer in each of the three models contained 17 neurons. In the hidden layers, the numbers of neurons were 30, 17 and 7 the 1-, 3- and 5-year models, respectively. The output layer in each of the three models had only one neuron representing the disease-free survival of HCC patients after hepatic resection.

The training group data were used to construct an ANN model, an LR model and a DT model. The ANN model included input, hidden, and output layers. Figure 1 shows the three independent ANN models for 1-, 3- and 5-year disease-free survival. The input layer in each of the three models contained 17 neurons: age, gender, liver cirrhosis, chronic hepatitis, AST, ALT, total bilirubin, albumin, creatinine, ASA classification, Child-Pugh classification, TNM stage, tumor number, portal vein invasion, biliary invasion, surgical procedure, and post-operative complication. In the hidden layers, the numbers of neurons were optimized using training and validation data in a trial-and-error process to maximize predictive accuracy [34], which resulted in 30, 17 and 7 neurons in the 1-, 3- and 5-year models, respectively. The output layer in each of the three models had only one neuron representing the disease-free survival of HCC patients after hepatic resection.

The LR model generates the coefficients for the following formula used for logit transformation of the probability of a patient having a characteristic of interest: 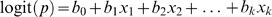 [23]. The formula 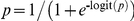 used for calculating the probability of the characteristic of interest in this study, where 1 = disease-free survival status and 0 = non-disease-free survival status.

Because of its easily interpreted decision rules, the DT model with C4.5 [22] was used for classification and regression. In this model, each object in the input dataset belongs to a class. Each object is characterized by a set of attributes (variables or predictors) that may have numerical and categorical (non-numerical) values. The goal of DT is to use a training dataset with known attribute-class combinations for generating a tree structure with a rule set for correctly classifying and predicting a similar test dataset. In addition to its root and internal (non-terminal) decision nodes, a DT has a set of terminal nodes (leaves), each of which represents a class. The rules associated with the DT, from the root to each terminal node (leaf), are easily interpretable for predicting a class. The steps of the learning process are (i) using an impurity function to select the most discriminative variable for data partitioning, (ii) repeating the partitioning until the nodes are sufficiently pure for use as terminal nodes, and (iii) pruning the completed tree to avoid over-fitting [41].

The software used to construct the ANN and DT models was Waikato Environment for Knowledge Analysis (WEKA) version 3.6.0 [42]. The LR model was constructed using SPSS for Windows version 6.1.

## Results

**Figure 2:**
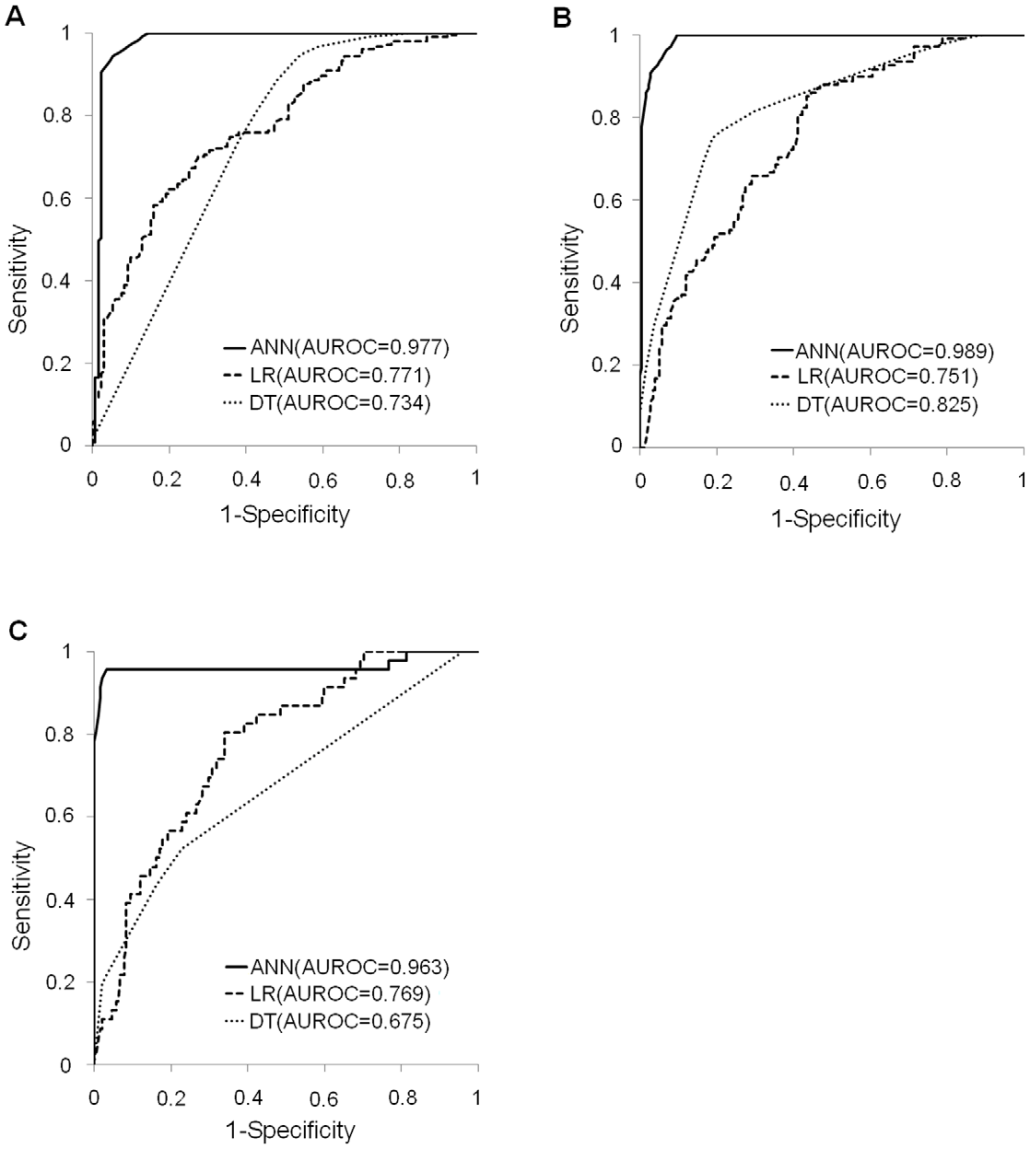
ROC curves and AUROCs for the 1-, 3- and 5-year disease-free survival models constructed for training groups using ANN, LR and DT. The AUROC values for 1-year (A), 3-year (B) and 5-year (C) disease-free survival were 0.977, 0.989 and 0.963 for ANN models, 0.771, 0.751 and 0.769 for LR models, and 0.734, 0.825 and 0.760 for DT models, respectively. In all disease-free survival models for training groups, AUROC values obtained by ANN were superior to those obtained by LR and DT.

**Figure 3:**
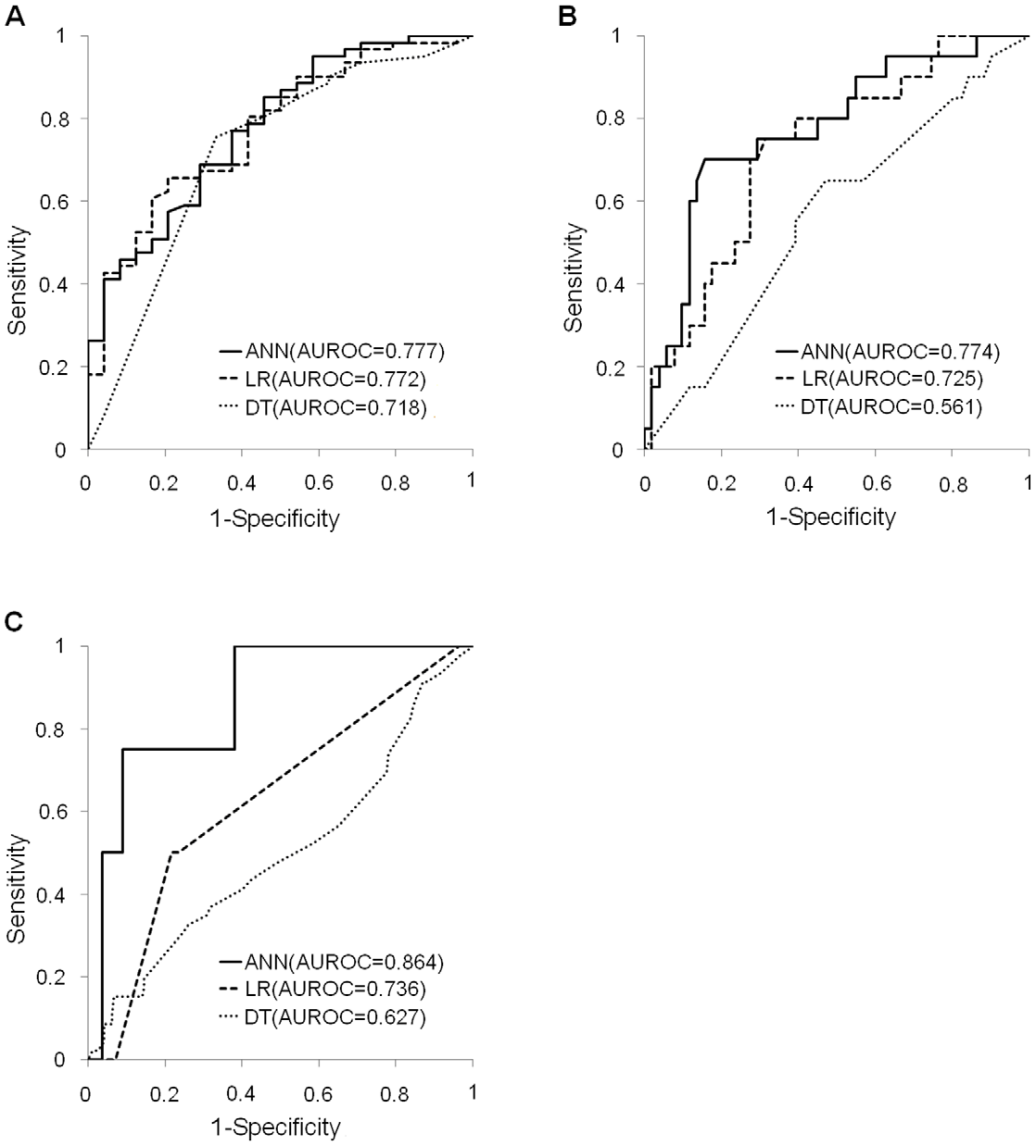
ROC curves and AUROCs for the 1-, 3- and 5-year disease-free survival models constructed for validation groups using ANN, LR and DT. The AUROC values for 1-year (A), 3-year (B) and 5-year (C) disease-free survival were 0.777, 0.774 and 0.864 for ANN models, 0.772, 0.725 and 0.736 for LR models, and 0.718, 0.561 and 0.627 for DT models, respectively. In all disease-free survival models for validation groups, AUROC values obtained by ANN were superior to those obtained by LR and DT.

For the training and validation groups, Figs. 2 and 3, respectively, show the receiver operating characteristics (ROC) curves for the 1-, 3- and 5-year disease-free survival models constructed using ANN, LR and DT. Tables 3 and 4 show the respective AUROC curves constructed using the data shown in Figs. 2 and 3. For example, the AUROCs for 1-year models constructed by ANN, LR and DT were 0.977, 0.771 and 0.734, respectively. For the training data and validation data, Tables 3 and 4 show the respective AUROC values, sensitivities and specificities for the 1-, 3- and 5-year disease-free survival models obtained by ANN, LR and DT. In the 1-year model for the training group, for instance, sensitivity and specificity were 0.962 and 0.916 when using ANN, 0.848 and 0.466 when using LR, and 0.948 and 0.458 when using DT, respectively. Notably, in all training groups and in most validation groups sensitivity and specificity for the 1-, 3- and 5-year models constructed using ANN were not only within acceptable limits, but were actually superior to those for models constructed using LR and DT.

**Table 3:** Performance comparison of ANN, LR and DT models for predicting 1-, 3- and 5-year disease-free survival in training groups.

	1-year(N = 342)			3-year(N = 283)			5-year(N = 238)		
	ANN	LR	DT	ANN	LR	DT	ANN	LR	DT
AUROC	0.977	0.771	0.734	0.989	0.751	0.825	0.963	0.769	0.675
Sensitivity	0.962	0.848	0.948	0.963	0.519	0.750	0.935	0.109	0.196
Specificity	0.916	0.466	0.458	0.931	0.789	0.811	0.979	0.958	0.979

**Table 4:** Performance comparison of ANN, LR and DT models for predicting 1-, 3- and 5-year disease-free survival in validation groups.

	1-year(N = 85)			3-year(N = 71)			5-year(N = 59)		
	ANN	LR	DT	ANN	LR	DT	ANN	LR	DT
AUROC	0.777	0.772	0.718	0.774	0.725	0.561	0.864	0.736	0.627
Sensitivity	0.787	0.754	0.885	0.700	0.450	0.550	0.750	0.000	0.000
Specificity	0.542	0.583	0.375	0.745	0.765	0.608	0.764	0.927	0.964

## Discussion

Model sensitivity and specificity are important when testing whether a model can accurately recognize positive and negative outcomes. Sensitivity and specificity must also be measured to determine the proportion of false negatives or false positives produced by a model [24]. Comparing false positive and false negative rates reveals the tendency of a model to misclassify positive patients as negative patients and vice versa [43]. The ideal model has both high sensitivity and high specificity [43]. In the current study, comparisons of predictive performance showed that the LR and DT models had poor sensitivity (<40%) but high specificity (>80%) for predicting 5-year disease-free survival in the training groups (Table 3); the DT model had poor specificity (<40%) but high sensitivity (>80%) for predicting 1-year disease-free survival in the validation groups (Table 4), and the LR and DT models had poor sensitivity (<40%) but high specificity (>80%) for predicting 5-year disease-free survival in the validation groups (Table 4). Specifically, Table 4 shows that the sensitivity values for predictions of 5-year disease-free survival with LR and DT models in the validation groups were zero. The explanation is the occurrence of false positives (i.e., type I error) [24]. That is, the LR and DT models, which had very low sensitivity, could be not used to screen for disease-free survival in HCC patients who had received hepatic resection since they lacked sufficient specificity for identifying true positives. However, sensitivity and specificity remained high in all ANN models (Tables 3 and 4). Since AUROC provides a superior performance index in addition to superior accuracy, AUROC was used to evaluate the predictive accuracy of classifiers [44]. The AUROC of a classifier can be defined as the probability of the classifier ranking a randomly chosen positive example higher than a randomly chosen negative example [44]. Therefore, the higher the AUROC, the higher the predictive accuracy [45]. This study also used AUROC values for performance comparisons of different prediction models. For the training groups, Table 3 shows that the AUROC values for 1-, 3- and 5-year disease-free survival were 0.977, 0.989 and 0.963 for ANN models, 0.771, 0.751 and 0.769 for LR models, and 0.734, 0.825 and 0.760 for DT models, respectively. In the validation groups (Table 4), the respective values were 0.777, 0.774 and 0.864 for ANN models, 0.772, 0.725 and 0.736 for LR models and 0.718, 0.561 and 0.627 for DT models. In all disease-free survival models, AUROC values obtained by ANN were superior to those obtained by LR and DT. Thus, the ANN models outperformed the LR and DT models in terms of predictive accuracy. The ROC curves in Figures 2 and 3 further show that the ANN was consistently more accurate in predicting 1-, 3- and 5-year disease-free survival compared to the LR and DT models, both of which demonstrated inconsistent results. The above comparisons thus confirm that ANN outperforms both LR and DT in predicting disease-free survival in HCC patients who have received hepatic resection.

Even when only seventeen easily obtainable parameters were used, the ANN models developed in this study demonstrated acceptable accuracy. Variables that were not significantly associated with disease-free survival were intentionally omitted when constructing the ANN models. The dependent variable indicates a decision by the lead surgeon in each case to perform a surgical intervention. In predictive mode, however, it can be considered a reliable estimation of confidence in the decision to operate on a specific patient since the ANN models were trained by a large patient database from teaching hospitals with highly qualified surgeons. Moreover, omitting this variable expanded the potential applications of the resultant model to circumstances in which advanced diagnostic.

Yeh et al. [10] used multiple logistic regression to predict associations between clinicopathologic factors and >5-year survival without recurrence in HCC patients treated with hepatectomy. Ercolani et al. [9] also evaluated prognostic factors affecting 5-year disease-free survival after liver resection in HCC patients with cirrhosis. However, the above studies [9], [10] focused on survival rates and predictors and did not compare the predictive accuracy of different statistical models. The current study, however, compared different statistical models in terms of accuracy in predicting 1-, 3- and 5-year disease-free survival after hepatic resection in HCC patients. The comparisons revealed that predictive accuracy significantly differed among ANNs, LRs and DTs. To our knowledge, very few studies have compared predictive performance in these three methods. The model comparisons showed that the ANN models of disease-free survival obtained superior AUROC values and have potential applications in decision support systems used to assess the need for hepatic resection in HCC patients.

In conclusion, comparison of prediction models for 1-, 3- and 5-year disease-free survival in HCC patients who have received hepatic resection revealed that the prediction models obtained by ANN machine learning method were superior to those obtained by conventional LR and DT. The AUROC values in the ANN models were generally higher than those in LR and DT models. That is, The ANN model had superior predictive accuracy. Therefore, this study demonstrated the feasibility of applying ANN in medical decision support systems that use clinical databases to predict disease-free survival in HCC patients who have received hepatic resection. Physicians may also consider machine-learning methods as a supplemental tool for clinical decision-making and prognostic evaluation.
